# Minimal Access Surgery in Neonates

**DOI:** 10.21699/jns.v6i3.614

**Published:** 2017-08-10

**Authors:** Ashrarur Rahman Mitul, Yogesh Kumar Sarin

**Affiliations:** 1 Department of Pediatric Surgery, Bangladesh Institute of Child Health and Dhaka Shishu (Children) Hospital, Bangladesh; 2 Department of Pediatric Surgery, Maulana Azad Medical College, New Delhi, India

## Abstract

Despite the significant advancement of minimally invasive surgery (MIS) in the adults and even in pediatric population, its role as the standard of care in the neonates has not yet been established among the pediatric and neonatal surgeons universally. Lots of controversies still arise though several advanced centers in the world having very experienced surgeons performing MIS for neonatal surgical conditions with promising outcomes. The unique physiological characteristics of a neonate make MIS quiet a challenging subject among these tiny babies. We have tried to look into the recent literature on the issues related to the use of MIS for the surgical management of neonates.

** INTRODUCTION**

"We are made wise not by the recollection of our past, but by the responsibility for our future" [GB Shaw].

Minimally invasive surgery (MIS) encompasses both laparoscopic and thoracoscopic procedures. MIS has gradually been accepted as a treatment modality for neonatal surgical conditions. In the hands of experienced surgeons, MIS has the added advantages of reduced tissue trauma, decreased pain medication, reduced hospital stay and better clinical information [1-3]. But these benefits of MIS do not come without costs, being both monetary and risk-based; and these risks rise disproportionately with the decreasing size of the patients, understandably, the neonates are most vulnerable. The surgeons have to face a daunting challenge, both mechanical and physiological in the management of neonatal surgical conditions [2-4]. Historically, pediatric surgeons have reservations to accept MIS as the standard of surgical care. The reasons being factors related to physiology, anatomy, anesthesia, technical and logistic simultaneously [2, 4]. A recent study involving pediatric surgeons from around the world has shown the skepticism among the pediatric surgeons about performing MIS for neonatal conditions. Just about one in 3 perform laparoscopic pyloromyotomy, and only 1 in 10 recommends this procedure for pyloric stenosis. Furthermore, fewer than 20% advocate laparoscopic Ladd's procedure for malrotation [5]. In this article, a review of literature was performed to address the uniqueness of MIS among the neonates, challenges faced and the ways to overcome those.

**MIS**

Minimal invasive surgical (MIS) techniques are used to perform operative procedures avoiding the morbidity of traditional open surgical wounds. Combination of technologies like the Hopkins rod lens system, insufflation devices allowing measured distension of body cavities with gas to provide the surgeon adequate space to work and miniaturization of video cameras to facilitate surgeon’s visualization inside the body cavities are the mainstay of MIS [6]. There are some other terminologies as well given to this procedure, like "key-hole surgery", "scar less surgery, "Minimally access surgery". Usually, MIS refers to laparoscopy (in the abdomen) and thoracoscopy (in the chest cavity). Endo-urological procedures are not discussed in this paper. MIS involves the use of telescopes attached to a camera and light source, trocars, long instruments, CO2 insufflators to make a working space inside the body cavity, and a modified surgical technique. The pediatric and neonatal instruments are further modified in an effort to be able work inside chest and abdomen of the tiny kids [2].

**Historical Background**

Stephen Gans was the first to report laparoscopy in pediatric surgery in 1971in his publication "Advances in Endoscopy of Infants and Children, where he used the term "peritoneoscopy" which actually was laparoscopy [7]. Gan's series also included a 2 kg, one-day-old baby with ascites, which was the first report of a laparoscopic procedure in a newborn [7]. Initially, laparoscopy was used for diagnostic purposes only. With the development of newer smaller instruments, MIS started to be used among very small babies as well. Klimkovich et al. described the first thoracoscopy in pediatric patients in 1971 after performing several procedures in this age group to diagnose mediastinal masses and cysts and lung parenchyma [8]. Alain reported first laparoscopic pyloromyotomy in 1991[9]. In the same year, Holocomb reported first laparoscopic cholecystectomy in a child[10]. Two years later, Rothenberg reported the first thoracoscopic lung lobectomy [2].in 1994-95, Willital created the premier pediatric MIS society IPEG (International Pediatric Endosurgery Group) [11]. Lobe and Rothenberg performed first thoracoscopic pure esophageal atresia repair in Berlin in 1999[12]. Next year, Georgeson [13] described first laparoscopic assisted anorectal pull-through. In 2001, Bax reported first duodenal atresia repair laparoscopically [14]. Rothenberg repaired first thoracoscopic tracheo-esophagela fistula in 2002[3]. Ponsky reported single port laparoscopy in children in 2009[2]. When very fine devices, precise movements and intelligent workarounds allowed surgeons to apply in MIS for neonates, the noble subfield of "Neonatal MIS" developed [2, 7, 12].

**Advantages of MIS**

There are definite evidence backed benefits of MIS even in the neonatal age group. MIS potentially reduces surgical trauma and fluid shifts which ultimately results in less postoperative analgesia; reduction in wound size also contributes. There is less bleeding as well [2,7]. Reduced wound size contributes to reduced wound infections, less chances of dehiscence and incisional hernias too. With proper instrumentation, heat loss should be less, which is a very vital factor for success in neonatal surgical procedures [2,15]. With the invention of digital cameras and sophisticated light sources, visualization and precision has become lot better with many times magnification; visibility at the corners with angled telescopes of body cavities has improved many folds than in open procedures [15]. Postoperative adhesions due to handling, talc, rubber or polyisoprene are less common [2]. Chest wall deformities like scoliosis have been eliminated with use of thoracoscopy after chest surgeries [2,16,17]. Early return to full feeds, early discharge from the hospital, with less post-operative morbidity are all factors contributing to the credit that MIS is deservingly attributed to [4]. There is less risk of postoperative ileus, and thromboembolism as well as reduction in nerve entrapment after an MIS[2,16].And last but not the least, power of exploration is an added advantage of MIS. Laparoscopic exploration can reliably diagnose malrotation, and can provide information that contrast often cannot. In inguinal hernias, visualization of opposite PPV with the same surgical trauma puts MIS superior to open herniotomy [2, 16].

**Challenges of MIS in neonates**

Despite MIS having several worth mentioning advantages over open procedures among neonates, pediatric and neonatal surgeons from around the globe historically have reservations in performing and recommending it for neonatal surgical conditions [2-4,16]. There are a number of challenges for MIS among neonates as well. There is loss of tactile sensation, loss of spatial and depth orientation in MIS [16]. With traditional instruments, the images are two dimensional rather than three [16]. Difficulty in the bleeding control and in extraction of tissue is among the technical negative issues deterring MIS among neonates too [16]. An operating instrument cannot be useful if it is longer than the patient, which is particularly true for in neonates MIS. The instruments are too long, too wide and just too big making no ergonomic sense [2,12]. For example, no 3mm, 20cm shear are available leaving surgeons the choice of using a blunt instrument longer than the patient or altering technique to incorporate different technology [2, 12]. Small instruments and a pediatric size 4mm, 20cm telescope still appear large compared with these small babies [2]. Clumsy MIS techniques are not minimally invasive. Ergonomics problems cause discomfort and fatigue for the surgeons, leading to imprecise movements with possibilities of surrounding organ injury [2]. Furthermore, difficulties in MIS are magnified in the small babies. The small endoscopes need to be positioned at a short distance thus limiting the field of view. Surface available for insertion of these instruments and the internal working place is limited so the tip of the instruments can move away of vision causing inadvertent injuries. Tremors and imprecise movements are exaggerated because of smaller extracorporeal and larger intra-corporeal length of instruments and magnification [4,12]. The abdominal surface to cavity ratio in neonates is less than that in adults and abdominal wall of them is very lax, as well as close proximity of structures putting trocar placement at risk [4,7,15,19]. Endosuturing and knot tying is one rate limiting step in neonates because of small reduced workspace. Introduction of digital 3D cameras, miniaturization of instruments like 3mm angled scopes, and "mini-laparoscopy", improved energy sources can prove important contributing factors for advancement of neonatal MIS [1,2,17]. Radially advancing STEP trocar technique can be useful to overcome problems due to laxity of neonatal abdominal wall [2]. Automated suturing devices have greatly simplified the suturing [2,15].

There is increasing concern about the effects of general anesthesia in neonates linking it to neurodevelopmental delay [1]. Some researchers have been trying to explore the feasibilities of regional and spinal block with sedation as well as performing multiple surgeries under single anesthesia to minimize the risks [1,19]. A recent international, multicenter randomized control trial reported use of Sevoflurane for less than 1 hour duration to be as safe as awake-regional anesthesia [1,19]. One lung ventilation in thoracoscopic cases is another challenging factor and anesthetist must be trained in and be aware of the procedure and how to overcome the awkward situations [4,17].

Newborns are simply not small adults [7]. Uniqueness of neonatal physiology poses specific challenges in performing MIS in this age group. The physiologic responses of pneumoperitoneum are more obvious than in adults.CO2 insufflation of the peritoneum have been found to cause hypercarbia, acidemia, and decreased oxygenation. Hypercarbia produces acidosis, decreased cerebral flow and other hazards. Hypothermia induced by CO2insufflation is a major concern as well. Trocars and other instruments leak CO2and some leaks may be relatively large. To compensate, surgeon must increase the flow. CO2isrelatively cool and dry. High-flowing gas cools patients, not from heat carried away by gas but from evaporative loss. Humidified worm gas and reduction of gas leak can attenuate this problem and its consequences [2]. Rise in intra-abdominal pressure is still a source of concern in neonatal physiology. The increased intra-abdominal pressure causes splinting of the diaphragm, which is the only muscle for respiration in neonates. Several pulmonary effects include diaphragmatic excursion and upward shift, reduced thoracic compliance and functional residual capacity; early closure of smaller airways and increased peak airway pressure, all leading ultimately to ventilation perfusion mismatch. Neuromuscular blockade, endotracheal intubation, adjustment of mechanical ventilation and PEEP are recommended. Raised intra-abdominal causes (IAP) increase in pulmonary and systemic vascular resistance, sudden bradycardia, reduced venous return and hypovolemia. These combined with lower head position increases intracranial pressure. Avoiding these factors may reduce intracranial pressure [19]. Published data suggests intra-abdominal pressure of 5-8 mmHg is tolerated well in neonates [4, 15].A newer concept of "gasless laparoscopy" eliminating the risks of pneumoperitoneum by using mechanical retraction may prove useful in future among neonates [19].

Positioning of table may need to be changed frequently during MIS; both trendelenburg and reverse trendelenburg positions are often used as well as left or right lateral tilt according to the surgical needs. Infants may be placed at the foot end of the table and secured properly. Well padding of the extremities should be ensured [12]. In neonates, periumbilical area should not be used for post placement because of the risks of puncturing the umbilical vessels [19].

Market-driven limitations in instrumentations remain an important issue in the development of neonatal MIS. The low volume of MIS procedures performed in the neonates acts as deterrent force to attract the manufacturing companies to come up with smaller, more sophisticated instruments [12,18,20]. Rothenberg has played an applauding role in convincing the manufacturing companies to design and produce miniature instruments so that now 2 and 3mm wide, shorter (18-20cm) instruments are available in the market, which can be used with safety in neonatal surgery [3,12,20]. A steep learning curve is another very important factor for acceptance and success of MIS in neonates [1,3,5,7,15,16,19].

**Indication and Contraindications**

Till date, quite a significant number of conditions are there where MIS is and has been used among babies <5 kg weight successfully [Table 1].

**Figure:**
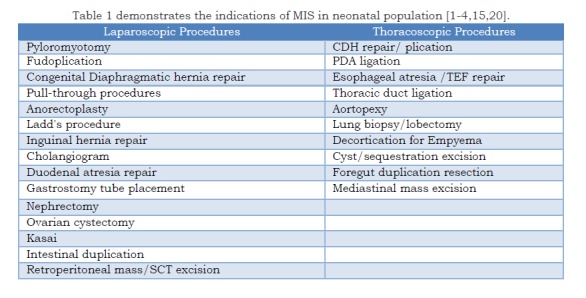
Table 1 demonstrates the indications of MIS in neonatal population [1-4,15,20].

With the advancement of MIS in neonates over the recent years, its contraindications in its use have become relative rather than absolute. Patients unsuitable for open surgery, uncontrolled bleeding diathesis and multiple previous abdominal surgeries are situations where MIS probably should not be tried. Babies with significant cardiac disease are at increased risk to tolerate MIS to compensate for the required physiological trauma already mentioned earlier [16].

**Complications**

A complication rates in pediatric laparoscopy have been estimated to be 0-67% being higher in babies weighing less than 5 Kg [1,16]. Nonspecific complications that can occur during any laparoscopic procedure are those related to pnemoperitoneum, those due to particular instruments like veress needle insertion, trocar placement. Specific complications occurring during particular types of surgery like injury to vas and vessels during inguinal hernia repair, injury to the vagus nerve during fundoplication etc. Postoperative nausea and vomiting can be is a significant consideration and should get priority attention to prevent aspiration [4,19].

**Discussion**

The "first do no harm" ethics of medical science has age long influence in medical practice and understandably more so when neonatal MIS is concerned [16]. Figuring out the challenges and difficulties in any particular specialty of science is not deterrent to its advancement, rather an infusing force to its development by overcoming these difficulties. MIS in the neonatal surgery has been safe, effective and provides the same benefits as its open counterparts [3]. But these successes have not been universal and trail leaders like Rothenberg, Holocomb, Georgeson and Lobe have played very substantial role in introduction of MIS in the neonates during last 20 years at most [2,3,12]. Adaptation of MIS among these small babies as the surgical default has been slow to develop worldwide, and only after the introduction of 2/3 mm instruments were made available, that too only in advanced centers [1,5,15,16]. In a series by Iwanka et al. complication rates after pyloromyotomy was as high as 9.7% and a meta-analysis by Hall and colleagues found that overall complications like mucosal perforations and incomplete pyloromyotomy was higher [1,18,21]. Whether MIS for congenital duodenal obstruction is superior to open approach still remains controversial evident from a very recent study. High leakage rate, anastomotic stenosis, missed distal duodenal obstruction makes open procedure still the operative procedure of choice [22]. Complex biliary reconstructions are challenging even in the hands of experienced laparoscopic surgeons. Increased recurrence rate is reported in case of laparoscopic inguinal hernia repair. The first multi-center, multi-surgeon review of esophageal atresia and TEF repair has shown results comparable to open thoracotomy, but the procedure itself has a steep learning curve and should be performed only by experienced MIS surgeons [18]. There has been increasing interest in attempting MIS for repair of congenital diaphragmatic defects in the neonatal period. But selection criteria for thoracoscopic repair are not well developed because of the effects of iatrogenic pneumothorax and its consequences; not all are good candidates for repair thoracoscopically and more studies are required to set up criteria. Furthermore, no consensus exists about which way to approach, thoracic or abdominal [18]. There are suggestions that despite the very rapid growth of MIS in the neonates, its application should not be considered as a direct alternative of techniques used in older children [4].

A very important issue is the involvement and interests of the manufacturers who produce MIS instruments for surgical use. Various 10mm and 5mm instruments used for adult MIS are the areas of concentration for the manufacturers because of the profits they make with increased volume of their use in adult population. That is the irony of present global open market economy all over the world and the neonatal surgeons are at their pity too. Manufacturers have been slow to produce products especially adapted to small babies. Many needs are still to be made. No 3mm, 20 cm shears, 3mm endoscopic clips are available till date compelling surgeons to use larger instruments [2,12]. Rothenberg must be credited for his extraneous efforts to be able to convince the industry, that they needed to produce these tools, and the key was that there was an adequate market so that it made financial sense [3,12].

The authors endorse the philosophy that, "Whatever is worth doing at all is worth doing well". Teamwork among surgeons and anesthetist is very vital; understanding the unique physiology and anatomy of a neonate; benefits and risks and knowledge of potential complications are key to success. It is also important that the parents do understand the same as well and a written informed consent is very important. The surgeon should never hesitate to convert to open procedure, which frankly is an evidence of his good judgment and not his incompetence. Surgical techniques should be tailored according to patients' need and not patients to the surgical technique [1,2,5,12,15]. The steep learning curve is well documented and should never be underestimated. Adequate training of the surgeons is mandatory for successful development of MIS among neonates. Coordination of neonatal surgeons and the manufacturers is vital in the development and production of miniature instruments [1,2,7,12,15].

Advancement of techniques and instrumentations should aid in the development of MIS among the neonates. Newer advances like Robotics in surgery looks promising in curtailing technical difficulties faced during traditional MIS. NOTES (Natural Orifices Endoluminal Surgery), and SILS (Single Incision Laparoscopic Surgery), mini-laparoscopy are now possible in the pediatric age and may prove successful in neonates as well. Newer, more sophisticated endosuturing devices, safer energy devices, slow-flow insufflators are all to provide safer MIS among neonates. Two mm instruments are now available in advances centers obviating the need for trocar insertion [2,5,12,15,17].

Any scientific innovation should be made with the objectives to address each and every one of nature's children, not for any particular class of people depending on economic abilities, class, race or creed. All surgical standards must be aimed to be equally beneficial and available to everyone globally, neonates are no exception. Similarly, neonatal MIS should be made available to every single neonate anywhere on earth; otherwise the innovation itself becomes discriminative and loses human values. We must never forget or ignore the philosophy as none other than Einstein said "“There is no great discoveries and advances, as long as there is an unhappy child on earth".

**Conclusion**

As is true for any other specialty of science and technology, MIS in neonates has to go through the stages of enthusiasm and disappointments. Appropriate infrastructures, availability of logistics, proper training of the pediatric and neonatal surgeons, careful patient selection and ability of the surgeons to identify difficulties early in the procedure by adding unfamiliar and uncomfortable techniques to their armamentarium. Introduction of finer and shorter instruments for use in the neonatal MIS is obligatory and the manufacturing companies should try to make profits by benefiting the health of the tiny babies. International cooperation is must for its development and acceptance universally. And, last but not the least, each and every neonate under the sky should have access to the benefits of MIS for their surgical conditions.

## Footnotes

**Source of Support:** None

**Conflict of Interest:** None
